# Gene–environment interaction of monoamine oxidase A in relation to antisocial behaviour: current and future directions

**DOI:** 10.1007/s00702-018-1892-2

**Published:** 2018-06-07

**Authors:** Kent W. Nilsson, Cecilia Åslund, Erika Comasco, Lars Oreland

**Affiliations:** 1 Centre for Clinical Research, Uppsala University, County Hospital, Västerås, Sweden; 2 Department of Neuroscience, Science for Life Laboratory, Uppsala University, Uppsala, Sweden; 3 Department of Neuroscience, Uppsala University, Uppsala, Sweden

**Keywords:** Antisocial personality disorder, Brunner syndrome, Conduct disorder, Genetic association studies, Gene–environment interaction, Genetic susceptibility, Juvenile delinquency, Monoamine oxidase A, Review

## Abstract

Since the pioneering finding of Caspi and co-workers in 2002 that exposure to childhood maltreatment predicted later antisocial behaviour (ASB) in male carriers of the low-activity *MAOA*-uVNTR allele, frequent replication studies have been published. Two meta-analyses, one in 2006 and the other in 2014, confirmed the original findings by Caspi and co-workers. In the present paper, we review the literature, note some methodological aspects of candidate gene–environment interaction (cG×E) studies and suggest some future directions. Our conclusions are as follows. (1) The direction of the effect in a cG×E model may differ according to the positive and negative environmental background of the population. (2) There is a predictor-intersection problem such that when measuring one type of maltreatment in a person, other kinds of maltreatment often co-occur. Other forms of abuse are implicitly considered in statistical models; therefore, it is difficult to draw conclusions about the effects of timing and the severity of different forms of stressful life events in relation to ASB. (3) There is also an outcome-intersection problem because of the major intersection of ASB and other forms of mental health problems. It is likely that the G×E with *MAOA* is related to a common unmeasured factor. (4) For the G×E model, in which the effect of the gene on the outcome variable is dependent on other predictor variables, theoretically, hypothesis-driven statistical modelling is needed.

## Introduction

The nature-versus-nurture debate has been ongoing since the time of ancient Greece (Aristotle 1984). Today, it is widely accepted that both genetic and environmental factors contribute to health and behaviour, and the theory of the “blank slate”—that the mind has no innate traits—has been criticized (Pinker 2003). However, despite the rather self-evident notions of nature and nurture, the difficulty obtaining proof of the concept without using a well-defined biological parameter (e.g., genetic make-up) has been noted repeatedly. To address this question, researchers such as Kidd and Matthysee (1978) and Bohman and co-workers (1982) have investigated twins reared together and have compared them with twins reared apart. Although twin studies are effective in distinguishing the influence of genes and environment, genes may also influence the environment, and conversely, the environment may influence gene expression (Moffitt 2005). For example, individuals may vary in their ability to cope with stressful experiences and environments depending on their genetic make-up (Craig 2007), a phenomenon commonly referred to as gene–environment interaction (G×E).

Among several phenotypes studied through the G×E lens, antisocial behaviour is particularly interesting and is the focus of the present review because many scholars have searched for the “warrior or worrier” gene. Heterogeneous neurobiological, psychological and behavioural components constitute aggressive behaviour. The association between cognition, emotion and aggression is well-known, and neural circuitries such as the serotonergic system have been shown to play a key role in regulating aggressive behaviour (Reif et al. 2007). On one hand, violence and aggressive behaviour represent an identifiable phenotype. When studying aggression, getting the phenotype right is essential for understanding the underlying mechanisms (Hodgins et al. 2009a). Different developmental subtypes of aggressive behaviour such as “life-course-persistent” and “adolescent-limited” have been described (Eley et al. 2003). On the other hand, aggressive behaviour has been described differently depending on the research field and theoretical framework (Hodgins et al. 2009a). Therefore, some researchers may be unaware of research using other labels of such behaviour, which in turn may obscure the understanding of these heterogeneous phenomena (Hodgins et al. 2009a).

Aggressive behaviour has a vast comorbidity or overlap with conduct disorder, antisocial personality disorder, alcohol use disorder, drug use disorder, major depression, anxiety and attention-deficit hyperactivity disorder. This complex overlap between these conditions has motivated interest in the common and differentiating mechanisms responsible for these co-occurring phenotypes.

In 1993, a non-sense mutation of the monoamine oxidase A gene (*MAOA*), which results in MAOA deficiency, was associated with a syndrome characterized by mild retardation, extreme reactive aggression, and violent and impulsive behaviour among males in a single Dutch kindred, the Brunner syndrome (Brunner et al. 1993). The association between *MAOA* and aggression was further recapitulated in animal models, in which male *Maoa*-knock-out mice demonstrate hyper-aggressive behaviour and heightened fear responses (Kim et al. 1997). As an enzyme, MAOA plays a major role in the metabolism of biogenic amines, including the neurotransmitters serotonin, norepinephrine and dopamine, which are involved in several brain functions associated with stress and regulation of emotion (Shih and Chen 1999; Shih et al. 1999a, b).

Humans and most other mammals produce the enzyme MAOA, which is encoded by a gene located on the X-chromosome, Xp11. In 1998, Sabol and co-workers identified a functional 30-bp variable number of tandem repeats (*MAOA*-uVNTR) in the promoter region of human *MAOA* (Sabol et al. 1998). The repeat is present in 2, 3, 3.5, 4, 5 or 6 repeats (R), which are associated with different effects on transcriptional and enzymatic activity (Deckert et al. 1999; Guo et al. 2008; Sabol et al. 1998; Huang et al. 2004). The most common alleles are those with 4R and 3R. Alleles with 3.5R or 4R are transcribed more efficiently than alleles with 2R or 3R, and are classified as high-activity (*MAOA*-H) and low-activity (*MAOA*-L) alleles, respectively (Deckert et al. 1999; Guo et al. 2008; Sabol et al. 1998). The transcriptional efficiency of the 5R allele is inconsistent in the literature because it has been classified as both a low-activity allele (Sabol et al. 1998) and a high-activity allele (Deckert et al. 1999). A more recent study reported that the transcriptional activity of the *MAOA*-uVNTR increases as a function of repeats, i.e., the 2R and 3R alleles is associated with lower transcription activity and the 3.5R, 4R and 5R alleles with higher transcriptional activity (Beach et al. 2010). No functional classification is available for the 6R allele. Because *MAOA* is situated on the X-chromosome, males have only one copy whereas females have two copies; therefore, females may be either homozygous or heterozygous.

With the demonstration that different alleles of the gene encoding MAOA show several-fold differences in enzymatic activity in transfected cell lines in vitro, researchers have become interested in investigating the genetic associations between this enzyme, psychiatric disorders and the G×E in both clinical and healthy populations. The first G×E study of human aggressive behaviour was reported by Caspi and co-workers in 2002 and showed that exposure to childhood maltreatment predicted later antisocial behaviour (ASB) in male carriers of the *MAOA*-L allele (Caspi et al. 2002). This innovative finding generated numerous replication attempts in the following years, with varying results. However, two meta-analyses, one in 2006 (Kim-Cohen et al. 2006) and the other in 2014 (Byrd and Manuck 2014), confirmed the original findings of Caspi and co-workers (Caspi et al. 2002) in males. The validity of the construct has been further supported by studies of rhesus macaques, which similarly to humans, carry orthologous high- and low-activity *MAOA* variants. Consistent with the findings in humans, a G×E with *MAOA* has been reported in rhesus macaques (Newman et al. 2005; Karere et al. 2009; Golub et al. 2016). As a new meta-analysis is currently being performed by Byrd and co-workers (personal communication), in the present review, we review the literature, discuss some methodological aspects of candidate gene–environment (cG×E) studies and suggest some future directions for research on the G×E with *MAOA*.

## Different theoretical frameworks in psychiatric cG×E research

Since 2002, when Caspi and co-workers presented their pioneering finding (Caspi et al. 2002), a steadily increasing number of cG studies have identified significant cG×E with psychiatric outcomes. However, the G×E research field in psychiatry has also attracted controversy and criticism, including criticism of studies of the *MAOA*-uVNTR genotype. A review of 103 studies of cG×E published from 2000 to 2009 suggested that the inconsistent findings of cG×E could be explained by differences in the design of the studies, statistical methodologies used, measurement of outcome variables and the included environmental factors (Duncan and Keller 2011). In addition, the dose and direction of the environmental factor being examined and cG×cGs are other possible contributors to the controversy (Comasco et al. 2013; Beaver and Belsky 2012; Boyce and Ellis 2005; Belsky and Pluess 2009; Nilsson et al. 2015). It is important to acknowledge that investigations of cG×E should include positive environmental factors (E-pos) even among individuals exposed to negative environmental factors (E-neg) (Keller 2014), i.e., the effects of both resilience and risk should be tested (Nilsson et al. 2015; Aslund and Nilsson 2018).

The cG×E research field has generally been dominated by the diathesis–stress framework, which assumes that certain genotypes increase the risk of adverse outcomes in stressful environments (Dick 2011; Manuck and McCaffery 2014). Research from the traditional diathesis–stress perspective has focused on genetic variations in the responsiveness to contextual adversity by comparing psychosocial risk with the absence of risk, but has neglected to measure the effects of a supportive environment or enriched care. Consequently, the diathesis–stress perspective has limited the results to interpretation of the investigated genes as “vulnerability genes”. In contrast to the diathesis–stress framework, the more recent differential susceptibility hypothesis suggests that cGs which interact with environmental events do not increase the risk of behavioural or psychiatric disorders per se; instead, they seem to alter an individual’s sensitivity to both the positive and negative influences in the environment (Belsky and Beaver 2011; Belsky et al. 2009; Belsky and Pluess 2009; Hankin et al. 2011). Similarly, the biological sensitivity to context model suggests that the G×E shapes an individual’s sensitivity to the environment over time and that some individuals have a high biological reactivity to both highly stressful and highly protective environments (Boyce and Ellis 2005).

Even though the last two theories emerged independently and differ regarding their definition of reactivity (relative to stress response in the biological sensitivity to context model) and sensitivity (relative to behavioural level in the differential susceptibility model), both make the assumption that environmental influences on developmental and life outcomes are moderated by neurobiological susceptibility to the environment (Ellis et al. 2011). Moreover, both theories highlight genetic variation in responsiveness to both adverse and supportive contextual conditions in a bi-directional manner (Ellis et al. 2011). According to the differential susceptibility perspective, those individuals who, according to diathesis–stress hypothesis, are especially vulnerable to adversity are simultaneously more likely to reap the benefits of supportive environmental conditions. In other words, some individuals are more generally susceptible than others to environmental influences, both for the better and for the worse (Belsky et al. 2007). Carriers of such genotypes who are reared in a positive environment show better-than-average positive outcomes, whereas carriers of the same genotypes reared in adverse conditions show negative outcomes compared with non-carriers (Reiss et al. 2013; Hankin et al. 2011). Such susceptibility effects have been shown in several cG×E studies that have examined both positive and negative environmental influences in relation to different cGs (van IJzendoorn et al. 2012; Hankin et al. 2011; Bakermans-Kranenburg and van IJzendoorn 2011; Oreland et al. 2007; Nilsson et al. 2006; Åslund et al. 2013; Roiser et al. 2007; Aslund and Nilsson 2018).

## Results of systematic reviews of *MAOA* G×E studies

### Previous meta-analyses

The first meta-analysis of interactions between the *MAOA*-uVNTR and childhood maltreatment in relation to ASB was published in 2006 (Kim-Cohen et al. 2006). This meta-analysis demonstrated that the association between child maltreatment and mental health problems, including ASB, attention-deficit hyperactivity disorder (ADHD) symptoms and emotional problems, was stronger in males who carried *MAOA-*L. Although several replication attempts of the original Caspi study reported negative findings, meta-analytical aggregation provided strong evidence for an increased vulnerability to childhood adversity among male carriers of *MAOA-*L in relation to ASB (Kim-Cohen et al. 2006).

The second meta-analysis was published by Byrd and Manuck in 2014 and included 27 original papers that investigated the interactions between *MAOA*-uVNTR and childhood maltreatment in relation to ASB (Byrd and Manuck 2014). This meta-analysis confirmed an association between cG×E and *MAOA*, and a higher probability of ASB among male carriers of *MAOA*-L who were exposed to childhood maltreatment. Byrd and Manuck also addressed the controversial question of cG×E with *MAOA*-uVNTR in females, which has been avoided in most studies because of the uncertain functionality of heterozygosity given the location of *MAOA* on the X-chromosome, as mentioned above. Consequently, most studies have excluded females or, in some cases, heterozygous females, from analyses. The meta-analysis did not show a significant cG×E in females. However, maltreatment alone predicted ASB preferentially, although weakly, in female homozygous carriers of *MAOA*-H (*MAOA*-HH), in contrast to the effects noted in males.

### Subsequent positive and negative findings

Since then, several studies have presented mixed findings (Table 1). Fergusson and co-workers, using a longitudinal design, showed a stable *MAOA*-L G×E, investigating a variety of adverse environmental and personal factors, in relation to three different antisocial behaviour among males from 15 to 30 years of age (Fergusson et al. 2012). To an increasing extent, researchers tend to include various complementary measures of both environmental factors and phenotypical outcomes while investigating the general principle of the link between environmental stressors and antisocial/aggressive/impulsive phenotypes, which contrasts with early attempts to define associations and identify thresholds for determinate forms of maltreatment or negative stress. Lavigne and co-workers highlighted the importance of expanding the range of psychosocial risk factors included in G×E studies to provide more specific models of different phenotypes (Lavigne et al. 2013). Consequently, several studies have reported both significant associations between measured factors and phenotypes, and non-significant findings for other factors or phenotypes. For example, a study of male prisoner inmates showed positive interactions between parent criminality and *MAOA*-L in predicting criminal behaviour, although the interactions between self-reported childhood abuse and *MAOA* was not significant (Armstrong et al. 2014). Another study of prisoners convicted of violent crimes showed associations between childhood physical neglect and *MAOA*-L in relation to lifetime aggressive behaviour (Gorodetsky et al. 2014). Among prisoners not exposed to physical neglect, the *MAOA*-L carriers were least aggressive, whereas among prisoners exposed to physical neglect, the *MAOA*-L carriers were more aggressive. However, no significant interactions in relation to impulsivity or hostility were found (Gorodetsky et al. 2014). A cross-sectional study by Zhang and co-workers (2017) tested G×G×E interactions between *MAOA*, the serotonin transporter gene (*5-HTT*) and sexual abuse in predicting aggressive behaviour in a sample of the adolescent Chinese general population. They reported a significant three-way interaction in which male carriers of *MAOA*-H and the short allele of the *5-HTT* had the greatest aggression tendencies when exposed to childhood abuse. A cross-sectional study of 507 Asian male adolescents found positive interaction effects between child maltreatment and *MAOA*-L in relation to aggressive behaviour (Zhang et al. 2016). A longitudinal study found positive associations of *MAOA*-L and parental punitive discipline at ages 1.5, 2 and 5 years in predicting male ASB, as measured by peer rating, self-report and official records at age 15–20 years among both Caucasian and African American men (Choe et al. 2014).

**Table 1:** Studies of *MAOA* gene–environment interaction in relation to ASB

Source, year	n	Male %	Age	Race	Study population	Study design	Environmental measure	Outcome measure	Finding of G×E	Risk/plasticity allele	Differential susceptibility effects
Zhang et al. (2017)	546	100	*M* = 15.6	Asian (Han nationality)	GP, middle school students	Cross-sectional	Childhood maltreatment, self-report	Aggressive behaviour, self-report	Partly positive: three-way interaction *5HTT* × *MAOA* × sexual abuse	*MAOA*-H and *5HTTLPR*-L	Not analysed
Massey et al. (2017)	285 children with their mothers	49.5	< 0–5	Mothers: 56.8% non-Hispanic Caucasian, 43.2% other races	GP	Longitudinal	Prenatal and concurrent stress exposure and tobacco exposure	Disruptive behaviour at age 5 years	Partly positive: three-way interaction of *MAOA* × prenatal stress exposure × sex	Boys: *MAOA*-H Girls: NS	Not analysed. Positive in illustrations
Galán et al. (2017)	187	100	1.5–22	53% Caucasian; 36% African American; 11% biracial/other races	GP	Longitudinal	Punitive discipline at age 1.5 years (laboratory task)	Hostility and aggression at age 10 years Violent attitudes at age 17 years. Official arrests, self-reported ASB at ages 20–22 years	Some positive two-way interactions Some NS interactions	*MAOA*-L	Not analysed
Smeijers et al. (2017)	142	100		Not reported	Forensic psychiatric outpatients with aggression problems	Retrospective/longitudinal	Structured childhood trauma interview. Aggression replacement training	Self-reported aggression. Treatment response	Negative	–	Negative
Watts and McNulty (2016)	3610	100	11–32	White (56%), Hispanic (15%), African American (18%), other (12%)	GP	Longitudinal/cross-sectional	Relationship with mother in wave 1 (age 11–17 years) interview	Criminal behaviour, wave 2 (age 13–19 years) interview Self-control in wave 1 interview	Negative two-way interactions Positive three-way interactions mother relationship × *MAOA* × *DAT1*	*MAOA*-L, *DAT1–10*R/10R Additive index of plasticity alleles	Partly positive
Zhang et al. (2016)	507	100	*M* = 15.81	Asian (Han nationality)	GP	Cross-sectional	Child maltreatment	Aggressive behaviour	Positive	*MAOA*-L	Not analysed
Holz et al. (2016)	125	57.6	25	European	GP	Longitudinal	Childhood life stress, age 4–11 years, parent interview	Activity in amygdala, hippocampus, and anterior cingulate cortex in a reactive aggression task	Partly positive: three-way interaction *MAOA* × childhood life stress × sex	Males: *MAOA*-L Females: *MAOA*-H	Not analysed Partly positive in illustrations
Nilsson et al. (2015)	1337	49.4	17–18	Scandinavian (85.3%), non-Scandinavian (14.7%)	GP	Cross-sectional	Sexual abuse, family conflict, positive child–parent relationship	Delinquency	Partly positive: two- three and four-way interactions *MAOA, 5HTT, BDNF*, sexual abuse, family conflict	*MAOA*-L	Positive
Choe et al. (2014)	189	100	1.5–20	African American (44%), Caucasian (56%)	GP	Longitudinal	Punitive discipline at ages 1.5, 2, and 5 years, laboratory task	ASB ages 15–20 years (peer rating, self-report, official records)	Positive: *MAOA*-L × punitive discipline for all outcomes	*MAOA*-L	Not analysed
Gorodetsky et al. (2014)	692	100	*M* = 40.4	Caucasian	Prisoners of violent crime	Cross-sectional	Childhood trauma, self-report	Life-time aggressive behaviour; self-report, interview, disciplinary records	Partly positive	*MAOA*-L	Not analysed Positive in illustrations
Armstrong et al. (2014)	94	100	*M* = 31.66	African American (59%), Hispanic (25%), White (12%), other (4%)	Prisoners	Cross-sectional	Childhood physical and emotional abuse (self-report), parent criminality	Criminal behaviour	Partly positive: parent criminality	*MAOA*-L	Not analysed Partly positive in illustrations
Haberstick et al. (2014)	4316	100	12–34	White (77.8%), non-white (22.2%)	GP	Longitudinal	Maltreatment at age < 12 years by retrospective self-report questionnaire at age 24–34 years	ASB composite index (behaviour, convictions and anger hostility at age 12–34 years), interviews and self-report	Negative	–	Not analysed
Byrd et al. (2014, personal communication)	18,652	59.3		Mixed	Mixed	Meta-analysis	Maltreatment	ASB	Positive	Males: *MAOA*-L Females: trend for *MAOA*-HH	Not analysed
Kiive et al. (2014)	373	59.0	15.6–24.7	European	GP	Longitudinal cohort study	Stressful life events, family environment, self-reports	Aggression (teacher report)	Negative	-	Not analysed
Lavigne et al. (2013)	175	55.4	M = 4.4	White	GP	Cross-sectional	Family psychosocial risk factors	Oppositional defiant behaviour	Negative	–	Not analysed
Gallardo-Pujol et al. (2013)	57	100	M = 22.77	Caucasian	College students	Experimental	Social exclusion task	Aggressive behaviour (laboratory)	Positive	*MAOA*-L	Not analysed
Hill et al. (2013)	209	49.8	< 0–5 weeks	Not specified	GP	Longitudinal	Prenatal stress exposure (life events and neighbourhood deprivation)	Infant negative emotionality (irritability)	Positive	*MAOA*-L, no sex differences	Not analysed. Positive in illustrations
Pickles et al. (2013)	193	Not specified	< 0–14 months	White British (96.1%), Other (3.9%)	GP	Longitudinal	Maternal sensitivity (laboratory)	Infant anger proneness	Positive: low maternal sensitivity × *MAOA*-L in males. High maternal sensitivity × *MAOA*-H in females	Males: *MAOA*-L Females: *MAOA*-H	Positive in illustrations
Verhoeven et al. (2012)	432	23.1	18–35	Western European descent	GP	Cross-sectional	Childhood trauma (self-report)	Aggression-related behaviours (self-report)	Negative (main effect of *MAOA*- in women)	*MAOA*-HH: main effect in women	Not analysed
Simons et al. (2012)	224	100	10–21	African American	GP	Longitudinal cohort study	Various hostile/demoralizing environments	Delinquency in 5th grade, aggression at age 20–21 years	Positive: cumulative plasticity index of *MAOA, DRD4, 5HTTLPR*	*MAOA*-L	Positive
McGrath et al. (2012)	192	0	*M* = 42.9	White	GP	Pregnancy cohort	Childhood physical maltreatment	Maternal problem behaviour/conduct problems	Partly positive (conduct problems)	*MAOA*-HH (females)	Not analysed
Fergusson et al. (2012)	399	100	0–30	White (88%), non-White (12%)	GP	Birth cohort, longitudinal	Various measures of childhood adversity	Criminal offending age 15–30 years. Convictions age 17–21 years	Positive	*MAOA*-L	Not analysed
Cicchetti et al. (2012)	312	100	M = 11.27	African American (67.1%), White (10.7%), Hispanic (18.2%), other (4%)	GP: low-income children	Cross-sectional	Childhood maltreatment (identified by the county authorities)	ASB	Positive	*MAOA*-L	Not analysed. Partly positive in illustrations

For a descriptive overview of the precedent studies, please see Byrd et al. (2014, personal communication) and Kim-Cohen et al. (2006)

Age is presented as mean (M) or range

*ASB* antisocial behaviour, *G×E* gene–environment interaction, *GP* general population, *H* high-activity variant, *NS* not significant, *L* low-activity variant, *HH* homozygous high-activity variant

However, several studies have also reported negative findings in recent years (Verhoeven et al. 2012; Kiive et al. 2014; Lavigne et al. 2013; Haberstick et al. 2014; Smeijers et al. 2017). Verhoeven and co-workers (Verhoeven et al. 2012) found no interaction between childhood trauma and *MAOA* in relation to aggression-related behaviours. Data were cross-sectional and self-reported by 18–35-year-olds. The study included both men and women, and a main effect of *MAOA*-HH was found in women in relation to aggression reactivity (Verhoeven et al. 2012). A longitudinal cohort study including both sexes found no interaction between *MAOA* and self-reported stressful life events or family environment in relation to teacher- or self-reported aggression in 18–25-year-olds (Kiive et al. 2014). Another longitudinal study in men found no interaction between retrospective reports of maltreatment (before age 12 years, self-reported at ages 24–34 years) and *MAOA* in relation to a composite ASB index, which included conduct problems, convictions for violent offences and disposition toward violence occurring between the age of 12 and 34 years (Haberstick et al. 2014). A recent study (Smeijers et al. 2017) investigated self-reported aggression and treatment responses in male forensic psychiatric outpatients with aggression problems. No interaction between childhood trauma, as determined in a structured interview, and *MAOA* was found in relation to self-reported aggression; although a main effect was reported in which male carriers of *MAOA*-L demonstrated more severe aggression. The authors found no differences in treatment response following aggression replacement training according to the *MAOA* allelic distribution (Smeijers et al. 2017).

In summary, consistent with the two meta-analyses discussed above (Kim-Cohen et al. 2006; Byrd and Manuck 2014), there is some support for the initial findings of a G×E with *MAOA*-L in relation to ASB among males from different settings, in different age groups and from different ethnicities. On the other hand, several studies have reported no G×E, although some have reported main effects of *MAOA* and indications of a sex difference in the direction and effect of *MAOA*-uVNTR.

### Sex differences in *MAOA* G×E studies

There are inconsistent findings of sex differences in the G×E with *MAOA* in relation to ASB. A few early studies on females reported interactions between *MAOA*-HH and environmental adversity in predicting female ASB, in contrast to the findings on *MAOA*-L in males (Sjoberg et al. 2007; Prom-Wormley et al. 2009; Åslund et al. 2011). These early findings were confirmed in a prospective study that investigated female problem behaviour, which found that *MAOA*-HH interacted with physical maltreatment to predict conduct problems (McGrath et al. 2012). However, other studies have reported that *MAOA*-L is also a risk allele in females, similar to that observed in males (Ducci et al. 2008; Enoch et al. 2010). The meta-analysis by Byrd and Manuck reported weak findings of an interaction between *MAOA*-HH and child maltreatment in predicting female antisocial outcomes, although the finding did not survive adjustment after removal of either of two study cohorts, and the interaction with general life adversities was not significant (Byrd and Manuck 2014). Sex differences are supported by some findings of three-way interactions of *MAOA*, environmental adversity and sex in relation to antisocial outcomes (Frazzetto et al. 2007; Massey et al. 2017; Holz et al. 2016).

### Infant and toddler *MAOA* G×E studies

Massey and co-workers (Massey et al. 2017) investigated prenatal and concurrent stress exposure in a longitudinal cohort study of children from infancy to 5 years of age. They reported a three-way interaction of prenatal stress, *MAOA* and sex in relation to disruptive behaviour at age 5 years. However, in contrast to most previous studies, boys with *MAOA*-H showed the highest levels of disruptive behaviour after having been exposed to prenatal stress. No significant interactions were found in girls. The authors speculated that testosterone levels associated with the pubertal transition in boys may alter the function or influence of *MAOA* on behaviour (Massey et al. 2017). However, a previous study on boys younger than 7 years identified the *MAOA*-L as a risk/plasticity allele, similar to findings in adolescents and adults (Kim-Cohen et al. 2006). A study by Pickles and co-workers (Pickles et al. 2013) showed distinct sex differences in 7-month-old infants, in which boys with *MAOA*-L showed less anger proneness when the mother had a high sensitivity toward her child, whereas girls with *MAOA*-H showed more anger proneness when the mother was highly sensitive (Pickles et al. 2013). By contrast, boys with *MAOA*-H and girls with *MAOA*-L showed little response to the environment, suggesting that there are sex differences in plasticity effects of *MAOA*-L in boys and *MAOA*-H in girls. However, another study from the same research group using the same study population found positive interactions between prenatal stress exposure and *MAOA* in predicting infant negative emotionality (irritability) in 5-week-old infants. A greater effect of increasing prenatal stress on negative emotionality was found for *MAOA*-L in both boys and girls (Hill et al. 2013). Similarly, a study by Enoch and co-workers (Enoch et al. 2010) showed interaction effects between *MAOA*-L and stressful life events (pre-birth–7 years) in predicting hyperactivity at age 7 years in both boys and girls. However, the findings were mixed in that family adversity did not interact with *MAOA* in relation to hyperactivity, and no cG×E associations between family adversity or stressful life events and *MAOA* were significant in predicting conduct problems at age 7 years. By contrast, Lavigne and co-workers (Lavigne et al. 2013) found no significant interactions between family psychosocial risk factors and *MAOA* in relation to oppositional defiant behaviour in a cross-sectional study of 4-year-old children. Conversely, the same study found significant interaction effects between family psychosocial risk factors and *MAOA*-L in relation to symptoms of depression and anxiety in boys (Lavigne et al. 2013).

In summary, studies of infants and toddlers to some extent show more variable results than studies of adolescents and adults. The various results when modelling different outcomes in the same populations complicates the interpretations. There seems to be a general plasticity pattern of *MAOA*-L in boys and *MAOA*-H in girls, and these different alleles appear to be more susceptible to negative environmental exposure.

### Considerations of differential susceptibility in *MAOA* G×E studies

The presentation of theories of differential susceptibility or genetic sensitivity (Belsky and Beaver 2011; Belsky et al. 2009; Belsky and Pluess 2009; Hankin et al. 2011) offered a possible explanation for the inconsistent findings of G×E with *MAOA*. Before these theories, studies had adopted a diathesis–stress perspective, which exclusively measures the effects of negative environmental factors such as childhood adversity in combination with *MAOA* in predicting the development of ASB. However, in 2007, we reported, “Among the boys, in predicting criminality, only presence of the low-activity allele significantly interacted with environment. In the boys carrying that allele, environment seemed to have a dual effect: in combination with a good environment it was protective against criminality….” (Oreland et al. 2007). These conclusions were drawn according to the apparent protective effect of *MAOA*-L observed in the illustrations presented in a previous report (Nilsson et al. 2006). In recent years, several studies have applied a differential susceptibility perspective by investigating both the cG×E of psychosocial risk and positive environmental factors.

In a longitudinal cohort study of African American males, Simons and co-workers (Simons et al. 2012) investigated differential susceptibility properties of 5-*HTT, DRD4* and *MAOA* in relation to delinquency and aggression. They used a composite index of various hostile and demoralizing environmental factors that predicted aggression in carriers of multiple plasticity alleles, including *MAOA*-L. Their illustrations include regions of significance analyses and show distinctly ascending differential susceptibility slopes with an increasing number of plasticity alleles.

A longitudinal study showed that maternal sensitivity predicted infant anger proneness in male infants with *MAOA*-L and female infants with *MAOA*-H (Pickles et al. 2013). The explained variance in the G×E models varied between the alleles and sex, with an approximate estimation of 11% for boys with *MAOA*-L, 2% for boys with *MAOA*-H, 0% for girls with *MAOA*-LL and 50% for girls with *MAOA*-HH. Plasticity effects were thereby seen in boys with *MAOA*-L and girls with *MAOA*-H, whereas boys with *MAOA*-H and girls with *MAOA*-L were mainly unresponsive to maternal sensitivity. Interestingly, the effect of the maternal sensitivity factor was opposite in boys and girls, i.e., low maternal sensitivity was associated with higher anger proneness in male carriers of the plasticity allele (*MAOA*-L), but with lower anger proneness in female carriers of the plasticity allele (*MAOA*-H).

A cross-sectional study by our group (Nilsson et al. 2015) investigated G×G×E interactions on *MAOA*-uVNTR, *BDNF* Val66Met and *5HTTLPR*. We found two-, three- and four-way interaction effects between genotypes, sexual abuse and family conflict in predicting adolescent delinquency. As predicted by the differential susceptibility hypothesis, carriers of the genotypes that would be expected to have the highest risk for delinquency in an adverse environment showed the lowest delinquency scores if the participants reported a positive relationship with their parents. Furthermore, high levels of positive child–parent relationships, even among children who experienced adversity, reduced the risk of delinquency.

Watts and McNulty (Watts and McNulty 2016) created another additive index of plasticity alleles by including the *MAOA*-L and dopamine transporter (*DAT1*) genotypes. They investigated the relationship between 11–17-year-old boys and their mothers in predicting criminal behaviour and self-control two years later. Watts and McNulty found that the effects of parenting on criminal offending and youth self-control were strongest among those who carried plasticity alleles for both genotypes. The effects were most pronounced in relation to poor parent–child relationships.

A study by Smeijers and co-workers (Smeijers et al. 2017) investigated male forensic psychiatric outpatients receiving treatment for aggression regulation problems. They found that males with *MAOA*-L and a history of multiple traumas had more severe levels of aggression. However, they found no significant G×E or support for any differential susceptibility effects of *MAOA*-L in relation to responsiveness to treatment for severe aggression.

It is possible that trends of differential susceptibility effects might be apparent when investigating the findings in general of studies to date that have explicitly applied the diathesis–stress approach. That is, the study aims did not include testing for differential susceptibility effects, and the study design did not include any positive or supportive environmental factors. Even so, such trends of differential susceptibility effects may be indicated in the statistical findings or graphical illustrations published in the literature. For example, illustrative graphs of interaction effects generally show an increased risk of ASB among male *MAOA*-L carriers who have been exposed to adversity or maltreatment. However, the same graphs may similarly indicate a lower risk of ASB among male *MAOA*-L carriers who have not been exposed to adversity or maltreatment. For example, several publications include illustrations indicating possible differential susceptibility effects of *MAOA*-L in men (Caspi et al. 2002; Kim-Cohen et al. 2006; Nilsson et al. 2006; Widom and Brzustowicz 2006; Frazzetto et al. 2007; Enoch et al. 2010; Wakschlag et al. 2010; Cicchetti et al. 2012; Hill et al. 2013; Armstrong et al. 2014; Gorodetsky et al. 2014; Holz et al. 2016) and of *MAOA*-HH in women (Holz et al. 2016; Prom-Wormley et al. 2009; Wakschlag et al. 2010). However, inconsistent patterns of possible sex differences in assumed allelic plasticity are also apparent (Enoch et al. 2010; Hill et al. 2013; Massey et al. 2017).

Additionally, some studies have reported a main effect of *MAOA* through which *MAOA*-L generates a decreased risk of ASB in males while simultaneously generating an increased risk of ASB in interaction with environmental adversity [e.g., see Kim-Cohen et al. (2006), Hart and Marmorstein (2009)]. By contrast, other studies have reported main effects through which *MAOA*-L is associated with a higher risk for ASB in males that is similar to the direction of the interaction effect (Armstrong et al. 2014; Smeijers et al. 2017).

In summary, suggestions of an epistatic interaction of *MAOA* and other related genes are supported to some extent by studies that have investigated the susceptibility properties associated with *MAOA*. Application of the differential susceptibility hypothesis to the interpretation of findings of both main and interaction effects may provide a possible explanation for the reported discrepancies in the direction of the effect in the susceptibility alleles. Several studies may be re-evaluated in terms of providing support for the idea of the susceptibility properties of *MAOA*.

### *MAOA* G×E in human experimental settings

The G×E with *MAOA* has been successfully reproduced in laboratory settings. Gallardo-Pujol and co-workers (2013) exposed participants to social exclusion in a laboratory computer task. Following provocation by social exclusion, male carriers of *MAOA*-L showed greater aggressive laboratory behaviour compared with *MAOA*-H carriers. Another study analysed interaction effects between *MAOA* and laboratory provocation in the form of exposure to a blast of noise, presumably pre-set by a bogus opponent, in predicting laboratory aggression (Kuepper et al. 2013). Carriers of *MAOA*-L exhibited substantially greater aggressiveness (blasting their opponent with noise) in reaction to high and extreme provocation trials, but there were no sex differences in the direction of effect. In a recent study, parental punitive discipline in a laboratory task at age 1.5 years interacted with *MAOA*-L in predicting male violent attitudes and ASB at 17 years (Galan et al. 2017). However, the same study did not show any significant interaction effects in relation to hostile attributional bias at 10 years or official arrests at 17 years (Galan et al. 2017).

Neuroimaging techniques represent excellent tools for disentangling the neural underpinning of G×E with *MAOA* in experimentally controlled settings. Initially, Meyer-Lindenberg and co-workers demonstrated that *MAOA*-uVNTR genotype-dependent differences in neural substrates are involved in processing emotions in healthy humans (Meyer-Lindenberg et al. 2006). *MAOA*-L was linked to reduced grey-matter volume in the cingulate gyrus, amygdalae, insula and hypothalamus in both males and females. Moreover, cortico-limbic activation during emotion regulation and cognitive control differed depending on the *MAOA* genotype (Meyer-Lindenberg et al. 2006). Greater left amygdala reactivity to emotionally arousing stimuli, together with lower activity of cortico-limbic regions, distinguished individuals with *MAOA*-L (Meyer-Lindenberg et al. 2006). Additionally, men carrying *MAOA*-L showed greater amygdala and hippocampus activity during recall of aversive information and lower dorsal anterior cingulate activation during response inhibition (Meyer-Lindenberg et al. 2006). Overall, the heightened amygdala-related emotional reactivity and reduced top-down regulation by prefrontal and pre-limbic areas profile *MAOA*-L carriers as more prone to both impulsive aggressiveness and mood disorders. The same researchers integrated the psychological predictors of emotion regulation in their later research to advance understanding of the biopsychosocial mechanisms affecting behaviour and mental health.

Holz and co-workers used functional magnetic resonance imaging (fMRI) to investigate the G×E and brain function in a high-risk sample of young adults. Male carriers of *MAOA*-L displayed greater activity in the amygdala and hippocampus during emotional face-matching of fearful/angry faces in proportion to the level of stress they were exposed to during childhood (Holz et al. 2016). These individuals were also characterized by lower inhibitory control, as assessed by decreased anterior cingulate cortex activity while performing a response inhibition task, which was related to the level of adversity experienced during childhood (Holz et al. 2016). By contrast, in this study, the *MAOA*-H variant seemed to be protective and was associated with less emotional reactivity and better inhibitory control. Interestingly, females showed the opposite pattern, i.e., activity in the hippocampus and amygdala increased with the level of childhood life stress in female *MAOA*-HH carriers, but decreased with the level of childhood life stress in female *MAOA*-LL carriers (Holz et al. 2016). This is the first evidence of an interaction between *MAOA-*uVNTR and stress on brain function in areas involved in emotional processing and aggression, and this study highlighted pronounced sex differences (Holz et al. 2016). Additionally, an earlier fMRI study of a single-nucleotide polymorphism of *MAOA* (rs6609275) found associations with brain activity in a network of frontal, parietal and occipital regions that correlated with working memory capacity and predicted externalizing symptoms in children (Ziermans et al. 2012).

Recently, *MAOA*-uVNTR was investigated in relation to dopamine release, as assessed using positron emission tomography and a radio ligand for the D_2/3_ receptors in males as they watched a movie with violent versus neutral content and later performed a laboratory aggression task (Schluter et al. 2016). Although subjects with *MAOA*-L self-reported greater aggression, they displayed no changes in dopamine release, but showed greater provoked aggressive behaviour after watching the neutral movie. By contrast, those with *MAOA*-H showed greater dopamine release in the dorsal and ventral striatum, as well as increased aggression after viewing the violent movie. Considering that people with *MAOA*-L reported to have been more frequently exposed to aggressive stimuli, Schluter and co-workers suggested that individuals genetically predisposed to aggression are less sensitive to already known provocative stimuli (Schluter et al. 2016).

### *MAOA* G×E in rodents

In addition to neuroimaging studies, preclinical models provide possible approaches for studying the neurobiological underpinnings of G×E with *MAOA* (*Maoa* in rodents). Consistent with the observed effects of *MAOA* mutations on ASB or aggression in humans (Brunner et al. 1993; Caspi et al. 2002), exposure to fearful experiences during the peri-pubertal period is used as a stress model to induce increased aggression during adulthood and to study the molecular effects in rodents (Marquez et al. 2013). Higher *Maoa* expression levels and acetylation of histone H3 on the *Maoa* promoter region have been found in the prefrontal cortex of male Wistar rats exposed to peri-pubertal stress compared with controls (Marquez et al. 2013). Pharmacological challenge with the MAOA inhibitor clorgyline reverses this behavioural pattern, thus demonstrating the involvement of MAOA in aggression (Marquez et al. 2013). The same authors found decreased prefrontal activity after social challenge in aggressive rats. This preclinical study elegantly provides pharmacological and molecular evidence for a role of MAOA in mediating the G×E in relation to aggressiveness. Preclinical evidence of the G×E with *Maoa* is scarce, but suggests *Maoa* as a potential moderator of the influence of the environment on brain and behaviour. Whether the association is consistent with the diathesis–stress, differential susceptibility or mismatch hypotheses remains to be studied. Similarly, the prospective and long-term molecular and behavioural outcomes of any interactive effect are unknown.

## Discussion

### Operationalization of ASB

It is unclear whether violence and ASB represent a coherent phenotype. The debate about whether nature or nurture is the origin of violence remains a topic among scholars who ask whether violence is a learned behaviour or a competitive biological mechanism that has been shaped throughout evolution. It has been questioned whether, because of the effects of testosterone on dominance and violence, males are designed for aggression and why boys in all cultures spontaneously engage in rough play. Children can display violent behaviour well before they have been exposed to war toys or cultural stereotypes, and the most violent age is toddlerhood not adolescence (Pinker 2003, pp 306–336). On the other hand, violence has decreased during the civilization process of human history because of increasing thresholds of shame and disgust in civilized culture (Elias 1939/2000), and this decline in violence is dependent on changes in our cultural and material milieu that have given peaceable motives the upper hand (Pinker 2011). Furthermore, homicide patterns show a closer relationship with income inequality than does mortality from all other causes combined, which is strongly related to social disorganization (Wilkinson et al. 1998). Feeling shamed, humiliated and disrespected might underlie the mechanisms of psychosocial processes linking inequality, violence, social cohesion and mortality (Wilkinson et al. 1998). It is also known that some individuals are more sensitive to shame, guilt and pride, which are related to violence, and that subcultures can reject or support violent behaviour (Gilligan 1996).

When studying ASB, getting the phenotype right is essential for understanding the underlying mechanisms (Hodgins et al. 2009a). A small group of individuals—5% of males and 1% of females (Farrington and West 1993; Moffitt et al. 2002)—commits 50–71% of all violent crimes (Moffitt et al. 2002; Hodgins 1994). Two distinct developmental subtypes of ASB have emerged: “life-course-persistent” ASB and “adolescent-limited” ASB (Eley et al. 2003). Hodgins and co-workers elucidated that ASB has been described differently depending on the research field and theoretical framework (Hodgins et al. 2009a). Within the medical or psychiatric research field, individuals with ASB are diagnosed as having early-onset conduct disorder (CD) followed by antisocial personality disorder (ASPD) in adulthood. In criminology, these individuals would have been labelled as life-course-persistent offenders (Moffitt et al. 2002). Such individuals might be described by personality researchers as being high on the externalizing spectrum (Krueger et al. 2005) and by psychological studies as being high on psychopathy traits, with a small number as presenting with the syndrome of psychopathy (Andershed et al. 2002). Because researchers often use the same labelling within their respective fields, researchers may be unaware of research using other labels of life-course-persistent ASB, which in turn might obstruct efforts to understand these phenomena (Hodgins et al. 2009a).

Results from twin and adoption studies have shown that life-course-persistent ASB demonstrates a higher heritability (≈ 60%) compared with adolescent-limited ASB (30–40%) (Eley et al. 2003). However, a shared environment is significant only for the adolescent-limited ASB and explains only 30–40% of the variance (Eley et al. 2003). Twin studies have been criticized for upwardly biased estimates that might contribute to the difference in estimated heritability between these two forms of ASB; therefore, a total population sibling-based or mixed design that uses both population-based sibling and twin designs has been proposed (Kendler et al. 2015, 2016). However, the explanatory effect derived from studies involving twins, adopted siblings and population siblings have important limitations in terms of G×E research because models of such designs often pre-suppose non-interaction effects and often lack the G×E term in their models (Plomin et al. 1977). Although this problem has been recognized since the 1970s, the research field has not incorporated such criticism, which adds confusion when interpreting the findings relating to the genetic versus environmental contribution to phenotypic expression.

Another important factor highlighted by Plomin and co-workers (1977) to current candidate G×E research is that one environmental factor that may explain a small percentage of the variance across all individuals may explain nearly all of the variance for a specific subgroup of individuals (Plomin et al. 1977). When entering an environmental factor and/or interaction term into a model, the coefficient of determination (“explained variance” or *R*^2^ of a model) and the change in *R*^2^ are important. However, the *R*^2^ estimate is a composite across all individuals and cannot describe the explained variance between individuals with different genetic and environment backgrounds. We believe that it is more important to compare the *R*^2^ between, e.g., carriers and non-carriers of a specific susceptibility allele when investigating the positive or negative interactions with a specific environmental factor.

Another important issue in future cG×E research is psychiatric comorbidity, which is affected by both genetic and environmental influences (Cerdá et al. 2010). This review by Cerdá and co-workers shows that genetic factors play a strong role in the relationship of comorbidity with major depression (MD) and generalized anxiety disorder or post-traumatic stress disorder, whereas genetic and non-shared environmental factors also make a moderate-to-strong contribution to the relationship between CD and substance abuse. They also found that several cGs, such as *5-HTT* and *MAOA*, as well as others involved in the function of the central nervous system, have been implicated in psychiatric comorbidity (Cerdá et al. 2010). For example, there is seldom a high explained variance in cG×E studies of depression that have investigated *5-HTT* promoter polymorphism (Munafo et al. 2009; Risch et al. 2009; Sharpley et al. 2014; Bleys et al. 2018; Culverhouse et al. 2017). By contrast, cG×E studies of aggression, delinquency or violence that have investigated *MAOA* polymorphism generally show larger effect sizes (Kim-Cohen et al. 2006; Byrd and Manuck 2014). A recent study showed that the susceptibility properties of the 5HTTLPR were distinctly less pronounced in relation to depressive symptoms compared to delinquency (Aslund and Nilsson 2018). These results are consistent with the results from the two original cG×E studies by Caspi and co-workers (Caspi et al. 2002, 2003).

If one assumes that the most common first primal reaction to emotional stress is aggression (Pinker 2003), a model of intersection phenotypes such as ASB, delinquency, criminality and violence should generate similar associations. A vast proportion of this association might be explained by hypothalamic–pituitary–adrenal (HPA) axis dysregulation, in which the nature of HPA disruption seems to be influenced by several environmental and individual factors including sex, age of onset of abuse, parental responsiveness, continued exposure to stressors or maltreatment, type of maltreatment, and type of psychopathology or behavioural disturbance displayed (Voorhees and Scarpa 2004). For some individuals, sustained exposure to stress may blunt the HPA axis response and development of depression (Briere and Jordan 2009). It is also noteworthy that the transition from exposure to emotional stressors, such as physical and psychological abuse, into the development of depression and anxiety is more pronounced among females than males and that not all exposed individuals demonstrate altered HPA axis physiology, which suggests that genetic variation can influence the consequences of trauma exposure (Briere and Jordan 2009; Neigh et al. 2009). Several studies have noted that *MAOA* is related to the aetiology of different mental illnesses, such as depression (Naoi et al. 2017). We suggest that the common factor for *MAOA* and other related genetic variations within the monoaminergic system (Iofrida et al. 2014) has a general impact on the social emotion regulation system, which in different studies may be conceptualized as depression (Beach et al. 2010; Naoi et al. 2017), impulsivity (Chester et al. 2015), alcohol consumption (Nilsson et al. 2007, 2008, 2010; Bendre et al. 2017), aggression and ASB (Kim-Cohen et al. 2006; Byrd and Manuck 2014).

There is a major intersection of ASB with criminality and other mental health problem phenotypes, which is often described as a high co-morbidity (Cerdá et al. 2010; Coker et al. 2014). However, both co-morbidity and co-occurrence of hierarchical patterns in the incidence of psychiatric symptoms are poorly understood. Symptoms that are rare in the general population are associated with the presence of many other symptoms or with other symptoms present to a severe degree in those with mental health problems (Sturt 1981). Because the research diagnostic criteria contained in the Diagnostic and Statistical Manual of Mental Disorders (DSM) (APA 2000, 2013) diagnoses in psychiatry are not defined at the etiological or pathophysiological level, research strategies to develop new diagnostic systems based on knowledge of the underlying neurobiological nature of disorders have been suggested (Andreasen et al. 1992). One way to handle the shortcomings in the diagnostic procedure is to use standardized rating scales that cover most of the whole spectrum of psychopathological symptoms in the DSM (Moller 2009).

When analysing the association of the *MAOA*-L variant in relation to ASB, because ASB has a huge overlap with CD, ASPD, alcohol use disorder (AUD), MD, anxiety and ADHD, one may question what is really estimated in the model. Similarly, if the ASB model is significant and one then tests for other outcomes, it may be possible to find associations of the G×E with *MAOA*-L in relation to CD, ASPD, AUD and perhaps MD, although possibly in the opposite direction or with the *MAOA*-H genotype. This is what has been found in a study exploring ASPD and MD in a model of *MAOA* and maltreatment, which showed that, in the context of child maltreatment, *MAOA*-H predisposes toward symptoms of MD whereas *MAOA*-L predisposes toward symptoms of ASPD (Beach et al. 2010).

It has become increasingly evident that the genetic architecture of psychiatric morbidity does not map onto the DSM (Stringaris 2013), in fact, cGs cut across DSM criteria (Thapar and Cooper 2013). This shows that the same genetic risks operate across diagnostic categories, which is also true for other risk factors (Thapar and Cooper 2013). Recently, the Research Domain Criteria (RDoC) project was introduced to develop new ways of classifying mental disorders based on dimensions of observable behaviour and neurobiological measures (Kaufman et al. 2015). *MAOA* codes for a protein that is involved in several biological processes, which are not fully understood. However, the G×E with *MAOA* findings in relation to ASB and various DSM diagnoses can be used to make future interpretations about the biological processes involved. The RDoC will continue to develop and its future work will show how the cG×E field fits into the RDoC framework. Some important questions are as follows. Could the G×E with *MAOA* be related to a common unmeasured factor not included in the DSM, perhaps impulsivity related to deficient social emotion regulation? Could such deficient social emotion regulation be a shared process involved in the phenotypes previously studied in *MAOA* G×E research? Could deficient social emotion regulation also reflect sex differences in the phenotypic response to social emotion threats? For the reasons noted above, cG×E research within the DSM-oriented field of psychiatry has a major outcome-intersection problem.

Finally, the literature has highlighted methodological issues regarding the measurement of ASB and the negative implications using different scales (Duncan and Keller 2011; Dick 2011; Dick et al. 2015). Although there is no universally accepted method to assess ASB or related phenotypes, some recommendations can be made. First, a questionnaire or semi-structured interview that assesses a large variety of such behaviours is warranted. Second, the “dosage” (i.e., estimation of the severity of the ASB) should also be measured. Third, the measurements should include estimations of the age at first occurrence and frequency, and whether the behaviour or event is still ongoing.

Several questionnaires and interview guides meet these criteria. From a cG×E point of view, it is important to measure the magnitude and severity of the behaviour or behaviours and to thereby discriminate between infrequent and mild ASB as opposed to more pronounced ASB. Scales always provide better discriminative power, and several different measurements could be recommended. On the other hand, such recommendations might favour the use of a specific instrument above others, although sometimes a particular instrument suited for a specific group of study participants or the use of multiple instruments may be warranted. Moreover, some scales might need to be modified according to the age and cultural aspects of the population investigated instead of strictly using a specific validated instrument that may not be suitable for a particular population.

Another issue relates to the use of different types of reports: self-reports, reports by parents, teachers or official records, face-to-face interviews or questionnaires, and retrospective or prospective studies. Among these types of reports, face-to-face interviews are thought to be more reliable than phone interviews or questionnaires sent by regular mail (Moffitt and Caspi 2014; Newbury et al. 2018). It is unclear whether gathering data by electronic questionnaires produces equal results as the use of paper questionnaires in terms of response patterns and response rates. It has been suggested that depression could be equivalently measured by internet and paper versions of two depression instruments (Beck Depression Inventory-II and Montgomery–Åsberg Depression Rating Scale, patient version) (Holländare et al. 2010). However, to our knowledge, there are no reports of such comparisons for ASB. *MAOA*-uVNTR may have a more general function in basic social emotion regulation and may thereby be related to many phenotypes associated with social emotion regulation. A similar concept was shown in a recent paper that describes emotional reactivity as a mediating mechanism for *MAOA* and childhood maltreatment and personality pathology (Byrd et al. 2018). We suggest that many different phenotypes and different measurements of such phenotypes must be examined using different methods and data reported by informants to gather information about each phenotype to elucidate as many sides as possible to understand the outcome-intersection problem.

### Operationalization of environmental factors

Childhood maltreatment and stressful life events present a multifaceted, intricate phenomenon. A review has proposed that the occurrence of abuse may be more important than the form, severity or duration of the abuse (Briere and Jordan 2009). It is unclear why specific forms of abuse correlate more in some studies than in others (Briere and Jordan 2009). Additionally, there is much commonality between maltreatment variables. Different maltreatment variables also occur in the context of and intersection with other markers of poor family function, such as witnessing domestic violence involving other family members, having an alcohol- or drug-abusing parent, low socio-economic status, low parental educational level or parental unemployment, living in a socio-economically deprived neighbourhood and having low social capital (Debowska et al. 2017; Patwardhan et al. 2017).

Consequently, if physical abuse is identified and measured in one participant, that participant will often also have experienced psychological abuse and/or neglect. For example, child sexual abuse is an uncommon incident that is almost always accompanied by other types of childhood maltreatment (Vachon et al. 2015). Therefore, even when measuring one kind of maltreatment in an individual, other kinds of maltreatment are often co-occurring, and such poly-victimization is often a neglected component of a child’s victimization experiences (Fisher et al. 2015). A common finding in most studies is that there are low-abuse and poly-victimized groups, and that multiple victimization is associated with the most adverse externalizing and internalizing outcome [for a review, see Debowska et al. (2017)]. However, many studies have reported a weak correlation between, e.g., sexual abuse and non-sexual abuse. This lack of strong association, along with similar statistical limitations in the identification of sex differences, has been elucidated by Vachon and co-workers (Vachon et al. 2015). In their study, sexual and non-sexual abuse were only weakly correlated. However, 89% of subjects who had been exposed to sexual abuse had also been exposed to non-sexual abuse, whereas only 9% of those who had been exposed to non-sexual abuse had been exposed to sexual abuse. Similarly, re-victimization is an often-ignored phenomenon. Fischer and co-workers (Fisher et al. 2015) reported that 37–55% of children who had been exposed to repeated episodes of domestic violence were frequently bullied, physically harmed or neglected and were later exposed to severe physical violence in adolescence.

In summary, every type of severe victimization in childhood seems to be broadly related to both the same and other types of severe victimization throughout adolescence. Such forms of childhood maltreatment have equivalent psychiatric and behavioural effects that extend from anxiety and depression to rule-breaking and aggression (Vachon et al. 2015). Consequently, the results from meta-analyses comparing specific contributions of different childhood traumas to adult outcomes might be questioned. For example, in one meta-analysis, Mandelli and co-workers (Mandelli et al. 2015) found that emotional abuse showed the strongest association with depression followed by neglect, sexual abuse, domestic violence and physical abuse (odds ratio 2.78–1.98). However, these findings would be expected based on the study by Vachon and co-workers (Vachon et al. 2015) because the vast majority of child maltreatment comprises emotional abuse and neglect, and these kinds of maltreatment have less frequent co-occurrence with other maltreatment forms. By contrast, almost all individuals who have been physically and/or sexually abused have also experienced emotional abuse or neglect (Vachon et al. 2015).

Therefore, many abused victims who have experienced abuse during childhood have had such experiences more than once and have also experienced, to a great extent, different forms of abuse. These victims are at greater risk of re-victimization in adolescence and adulthood. Because of the cumulative effects of different aspects of childhood trauma, the co-existence of these traumas and the direct relationship between severe abuse and higher rates of co-occurrence, determining the specific association between one form of childhood abuse and consequences in adulthood is problematic (Briere and Jordan 2009; Vachon et al. 2015; Fisher et al. 2015; Debowska et al. 2017). Therefore, a variable that defines one form of childhood abuse will often concurrently index other forms of abuse, and those other forms of abuse are thereby implicitly considered in statistical models. Additionally, there is also a cumulative additive family risk of child maltreatment in that the combined effects of socio-economic disadvantages such as low parental income, unemployment and housing instability, as well as parental characteristics such as mental and physical health, use of alcohol and domestic violence elevate the risk (Patwardhan et al. 2017). Therefore, in our first cG×E studies, we used the “type of residence” (multifamily housing or single-family home) as a proxy variable for low socio-economic status or risk milieu. We found that, in relation to most ASB variables, this proxy variable interacted more strongly with *MAOA*-uVNTR compared with maltreatment/assault among boys and sexual abuse among girls, (Nilsson et al. 2006; Sjoberg et al. 2007).

A variable that measures recently experienced trauma will often include the effects of previously experienced trauma, as a result of re-victimization, and thereby it can be difficult to draw conclusions about the effects of the timing and severity of different forms of stressful life events in relation to ASB. Moreover, variables that predict maltreatment or in other ways predispose toward stressful life events, such as low parental income, unemployment or living in a poor neighbourhood, might increase the risk for maltreatment exponentially. Therefore, some individuals may experience a high dose of many stressful life events, including trauma, which may not always be explicitly measured. As seen in previous reports, there are clear indications of a dose–response pattern of the environmental load, which shows stronger interaction effects when combining more than one adverse life event. This predictor-intersection problem may be intractable when using standard regression methods because of issues of multicollinearity. A possible solution, in addition to studying them in a traditional adjusted model, may be to summarize them into a single measure using principal component analysis or a composite index of stressful life events and to use penalized regression methods such as ridge regression. However, there is a need to develop new analytic strategies to handle this predictor-intersection problem.

When it comes to differentiating the diathesis–stress model from the differential susceptibility models, there are some important protective factors against ASB. Garmezy and Rutter (1983) suggested attributes that may provide protective environmental or resilient factors: first, individual attributes such as good intellectual skill, positive temperament, and positive views of the self; second, family attributes such as high levels of warmth and cohesion within the family, high expectations and parental involvement with the youth; third, community attributes such as good schools, neighbourhood resources and strong social networks.

However, in the discourse about risk and resilience factors, many scholars have objected that the above suggested resilience attributes equate to previously described risk factors but have been simply observed from another perspective (Luthar and Zelazo 2003). Therefore, the question is whether the E-pos and E-neg factors lie on the same continuum or whether they are qualitatively different. This has been a topic of passionate debate in the literature on resilience (Coleman and Hagell 2007). Another much debated topic is whether E-pos factors should be seen as resilient factors in general, such that all individuals, both those who are seriously affected by E-neg life events and those who have not experienced any E-neg life events, benefit equally, or whether specific E-pos factors are relevant to adversity only, or even act only among individuals with specific E-neg factors. For a review of the adolescent resilience field see Coleman and Hagell (2007), and Luthar and Zelazo (2003). To our knowledge, these questions have been more or less overlooked in the field of cG×E research, both within the traditional diathesis–stress perspective and the differential susceptibility perspective.

Positive family factors have long been interlinked with resilience to ASB. The family is an important influence through factors such as parental control, parental support, family harmony, responsibility for chores or required helpfulness, security and stability, family norms and morality (Homel et al. 1999). We believe that such family factors might be a good place to start when investigating the genetic plasticity properties in relation to individually differentiated environments (Aslund and Nilsson 2018). We further believe that the research field on adolescent resilience to ASB and other mental health problems will benefit from models that take different biological aspects into account, given that, for example, *MAOA* has a profound effect on ASB in those individuals who carry the plasticity alleles (Caspi et al. 2002), and have experienced different degrees of both E-pos and E-neg life events.

Furthermore, we believe that the newly introduced and commonly used methods of including both the negative and positive aspects of the environmental load in the same variable (from very poor to very good parenting), as suggested in analysis of region of significance (RoS), have some shortcomings because such models cannot estimate the effects on resilience of a positive environment in individuals exposed to different degrees of other negative environments. A simple recently presented model (Aslund and Nilsson 2018) has revealed two things. First, that there is a significant sex difference in cG×E, which might be missed when using sex-separated models, and second, the importance of introducing the final interaction term when analysing sex×cG×E-neg×E-pos, which sets the effects for all lower-order interaction terms.

Future studies using multivariable models are needed to investigate E×E interactions, including both negative and positive environmental factors (E-neg×E-pos) to attend to what we here propose as the predictor-intersection problem.

### Global and cultural variations in ASB

There is a vast difference in the prevalence of mental health problems, aggression and the proportions of victimized individuals in different countries and regions of the world (WHO 2002, 2010). These have important implications for investigating both the predictor-intersection problem and outcome-intersection problem in different regions. Cultural violence is defined as any aspect of a culture that can be used to legitimize violence in its direct or structural form (Johan 1990). Cultural acceptance of violence as a conflict-resolving mechanism or as a child-rearing method is a risk factor for all types of interpersonal violence (WHO 2002). Such cultural aspects of violence may also partly explain why countries that experience high levels of one type of violence also experience high levels of other types of violence (Lansford and Dodge 2008).

It is unclear whether *MAOA*-uVNTR is strongly associated with violence or whether another variable can be operationalized to explain aggression or violence. It is also unclear whether such genetic variations have a common biological and evolutional advantage by helping the individual cope in different environments. There are some clues from earlier studies. Studies from societies with a low rate of violence, such as Sweden, have shown a stronger association of a G×E with *MAOA* with non-violent ASB (e.g., stealing or vandalism indexes) compared with violent ASB (violence index), although the strongest model was a total ASB index that included stealing, vandalism and violence (Nilsson et al. 2006; Sjoberg et al. 2007). One may speculate that, in a more pro- or anti-violence culture, a model of the interaction between *MAOA*-uVNTR and the environment will generate different results for different measurements of ASB in different countries and in different subcultures.

### Sex differences in the G×E with *MAOA*

One problem with studying the interaction of phenotype with ASB is that, if almost all individuals with a specific disorder or behaviour are male but very few males have the disorder, then sex might not be found to correlate with the disorder (Vachon et al. 2015). Therefore, in studies of depression and/or criminality, which are disproportionally distributed between males and females, and where a sex difference in genetic contribution is suspected, the conclusions from analyses of sex-separated models or combined-sex models without the inclusion of a sex interaction term can be questioned.

Another problem in the cG×E approach to studying the influence of *MAOA* is the location of the gene on the X-chromosome. Females have two X chromosomes, but males have only one and heterozygosity may be present in females but not in males. Because *MAOA* expression in heterozygous allele carriers is unclear, many investigators have selected only males or have eliminated heterozygous females from their samples (Ficks and Waldman 2014). Such unknown heterozygous effects have; however, been analysed for other cG×Es, such as the serotonin transporter gene *SLC6A4*, and the *5HTTLPR* polymorphism in particular. If one expects the heterozygous individual to be intermediate in phenotypic expression between the two homozygous forms, one will not consider molecular heterosis. Such molecular heterosis occurs when a person heterozygous for a specific genetic polymorphism shows a significantly greater or lesser effect for a phenotype than does someone homozygous for either allele (Comings and MacMurray 2000). Accumulating evidence shows that molecular heterosis is common in humans, may occur in up to 50% of all gene associations and is important for genes within the monoaminergic system such as *DRD1, DRD2, DRD3, DRD4, HTR2A* and *SLC6A4* (Comings and MacMurray 2000). If such a heterosis effect exists, it will be masked using a method that includes homozygous individuals with the susceptibility allele into the same group as those with one susceptibility allele compared with individuals homozygous for the other non-susceptibility allele. Therefore, one may question the approach to analysing *MAOA* in females by including only those who are homozygous for the short or long allele, thereby excluding heterozygous individuals, or by defining heterozygous females as those with the risk allele, compared with individuals homozygous for the non-risk allele [for a review, see (Byrd and Manuck 2014)].

One hypothetical explanation to the sex difference was published by Sjöberg and co-workers (2008). They reported that an interaction between the *MAOA* genotype and cerebrospinal fluid testosterone concentration predicted ASB and suggested that this interaction may be mediated by a direct effect on gene transcription (Sjoberg et al. 2008). Testosterone level may affect the transcription capacity of *MAOA* via binding at androgen response elements or transcription factors Sp1 (activator) and R1 (suppressor) in the promoter region (Ou et al. 2006). In addition, testosterone or its aromatized form, oestradiol, has been suggested to interact with MAOA metabolites such as dopamine, serotonin or norepinephrine (Chaudhari Nirja and Nampoothiri Laxmipriya 2017; Belelli et al. 2006; Zheng 2009).

Ten years after this interaction between *MAOA* and testosterone was proposed, a novel double-blind randomized experiment was conducted involving healthy males, who were given either a placebo or testosterone as a topical gel containing 50 mg testosterone. This study found that, after testosterone administration, *MAOA*-L carriers displayed greater risk-taking behaviour (Wagels et al. 2017). Oestrogen and progesterone administration have been shown to decrease *MAOA* mRNA expression in female rhesus monkeys (Gundlah et al. 2002). Future research will probably elucidate the functionality of *MAOA* in the primal environment, particularly the effects of the individual’s sex in determining the effect of *MAOA*. Because neither the predictors (maltreatment and abuse) nor the phenotypic outcomes are independently distributed between males and females, there is a need to consider sex in future studies. Moreover, if there is a sex-dependent cG×E with the phenotypes studied, both the main effect and interaction effect of sex should be included and adjusted for (Keller 2014). Consequently, in the case of sex differences, sex-separated analyses will not show the full properties of the cG×E studied, but may suggest the sex-specific direction of the effects.

### Functionality of *MAO* with different number of repeats

It is generally recognized that transcriptional activity increases as a function of the number of variable tandem repeats, i.e., the 2R and 3R alleles result in lower transcriptional efficiency and the 3.5R, 4R and 5R alleles result in higher transcriptional efficiency (Deckert et al. 1999; Guo et al. 2008; Sabol et al. 1998; Huang et al. 2004; Beach et al. 2010). Based on these findings, a general nomenclature for the gene is used to describe the associations with low or high transcriptional efficiency of the *MAOA* promoter, which are abbreviated as *MAOA*-L and *MAOA*-H. However, the activity of MAOA in the adult brain has repeatedly been shown to be poorly associated with genotype (Fowler et al. 2007; Alia-Klein et al. 2008a, b), which argues for a possible developmental effect of the functional polymorphisms of *MAOA* on neurocircuits through the regulation of embryonic/foetal serotonin levels. This interpretation is supported by studies showing disturbances of the same cortico-limbic structures resulting from the absence of the serotonin transporter during development (Wellman et al. 2007; Bearer et al. 2009).

Serotonin has been shown to modulate the outgrowth of terminals from serotonergic neurons both directly and indirectly in an auto-regulatory feedback loop [for review, see Whitaker-Azmitia (2005)]. This negative feedback loop seems to be dependent on the 5HT1A receptor, which is expressed early during development. In summary, excessive serotonin levels during brain development may negatively affect both the size and functional capacity of the serotonergic system itself. This may in part explain the paradoxical relationship between genetic variants associated with an increased level of available serotonin (low-functioning alleles of *MAOA* and *5-HTT*) and the link to behavioural traits and psychiatric disorders associated with lower levels of serotonin in the brain and its metabolite 5HIAA in cerebrospinal fluid. In other words, the low-functioning variants of *MAOA* and *5-HTT* may be associated with an increased risk of psychiatric disorders because of increased levels of serotonin during central nervous system development, which cause functional alterations to the neurocircuits critical for emotional processing while simultaneously inhibiting the outgrowth of the serotonergic system.

The hypothesis described above is consistent with findings of an effect of *MAOA* genotype on sensitivity to the environment that is apparent at a very early age (see the “Infant and toddler *MAOA* G×E studies” subsection above) and has been discussed in detail by Nordquist and Oreland (2010). Such a view would also explain the well-documented observation that personality traits are stable during the entire lifetime and that personality traits in which serotonergic “capacity” are involved also are associated with sensitivity to environmental factors.

No studies of the cG×E with *MAOA* have investigated the functionality. The investigated associations are always based on the number of allele repeats. The questions that have not been asked are what happens to the transcriptional activity of *MAOA* in different environments and to what extent is there a difference between different *MAOA* gene alleles regarding proneness for epigenetic changes in males and females. For example, among male *MAOA*-L carriers who have experienced maltreatment, more alcohol-related problems are found as a moderated effect if the individual displayed lower *MAOA* methylation levels of CpGs 13–16 in the first intron compared with to H allele carriers (Bendre et al. 2017). Moreover, a new problem can appear when studying epistatic effects (cG×cG or cG×cG×E) as aggregated genetic risk scores (AGRS) in association with a phenotype (Stoltenberg et al. 2012; Nilsson et al. 2015). If the genetic combination results in a low transcriptional activity both on the serotonin transporter gene *SLC6A4* and on the *MAOA*-uVNTR, it is unclear to what extent monoaminergic turnover is affected.

New technologies, such as studying organoids (Camp et al. 2015; Quadrato et al. 2017), might elucidate the functionality of *MAOA* through cortical development (Camp et al. 2015), including the formation of dendritic spines and active neuronal networks (Quadrato et al. 2017). Such new technologies might allow one to test the individual effects of sex, epistatic factors and environmental effects on the functionality of genes.

### *MAOA*-uVNTR: a possible susceptibility gene

If the differential susceptibility theories of cG×E are correct, the outcomes of previous studies of cG×E from the traditional diathesis–stress perspective would be expected to vary depending on the environmental measurements in the study population. For example, the failure to measure positive environmental influences that might counterbalance adversity in susceptible individuals could lead to false-negative findings of cG×E studies that apply the diathesis–stress model. The inconsistency between studies is frequently debated in this research field (Byrd and Manuck 2014; Munafo et al. 2009; Risch et al. 2009; Duncan and Keller 2011). Moreover, the existence of differential susceptibility effects would mean that meta-analyses of cG×E effects in diathesis–stress studies run the risk of producing null findings depending upon the extent to which the protective effects of the positive psychosocial factors were implicitly included, but not measured in the “no-stress” environment arm of the study populations. In future studies, it will be important to describe both positive and negative environmental factors because they are unevenly distributed in the population. Studies of ASB in clinical or other psychiatric samples explicitly have a higher proportion of negative and a lower proportion of positive environmental loads compared with unaffected control samples (Hodgins et al. 2009b; Larm et al. 2010). Therefore, it is crucial for the future of cG×E research in psychiatry that environmental influences be defined in terms of both positive and negative effects, and that these are measured accordingly.

In 2006 and 2007, two studies from our group showed distinct susceptibility effects of *MAOA* among boys carrying the L-allele. We reported a dual effect showing that adolescent males carrying *MAOA*-L showed greater levels of delinquency when reared in an adverse family environments and lower levels of delinquency when reared in a positive family environment (Oreland et al. 2007; Nilsson et al. 2006). However, at that time, we as well as others applied the diatheses–stress framework in the interpretation of the results. By studying the graphs published in previous studies that also applied the diathesis–stress model, we indeed found indications of a decreased risk of ASB among males with *MAOA*-L with no adverse environmental exposure (Caspi et al. 2002; Kim-Cohen et al. 2006; Widom and Brzustowicz 2006; Frazzetto et al. 2007; Enoch et al. 2010; Wakschlag et al. 2010; Cicchetti et al. 2012; Hill et al. 2013; Armstrong et al. 2014; Gorodetsky et al. 2014; Holz et al. 2016). Simons and co-workers (Simons et al. 2012) and Watts and McNulty (2016) investigated AGRS, including *MAOA*, and reported results that are consistent with the differential susceptibility theory. Moreover, in a longitudinal study, boys with *MAOA*-L and girls with *MAOA*-H showed plasticity effects, although low maternal sensitivity was associated with higher anger proneness in male carriers of the plasticity allele but with less anger proneness in female carriers of the plasticity alleles (Pickles et al. 2013). In addition to sex differences, in an epistatic model that did not use AGRS, distinct susceptibility effects related to *BDNF, 5HTTLPR* and *MAOA* genotypes were found, both in interactions with each other and with positive and adverse environments in relation to adolescent delinquency (Nilsson et al. 2015). Furthermore, carriers of the susceptibility variants of the genotypes who had experienced family conflicts and/or sexual abuse also exhibited a protective effect from a positive parent–child relationship in relation to delinquent behaviour (Nilsson et al. 2015).

**Fig. 1:**
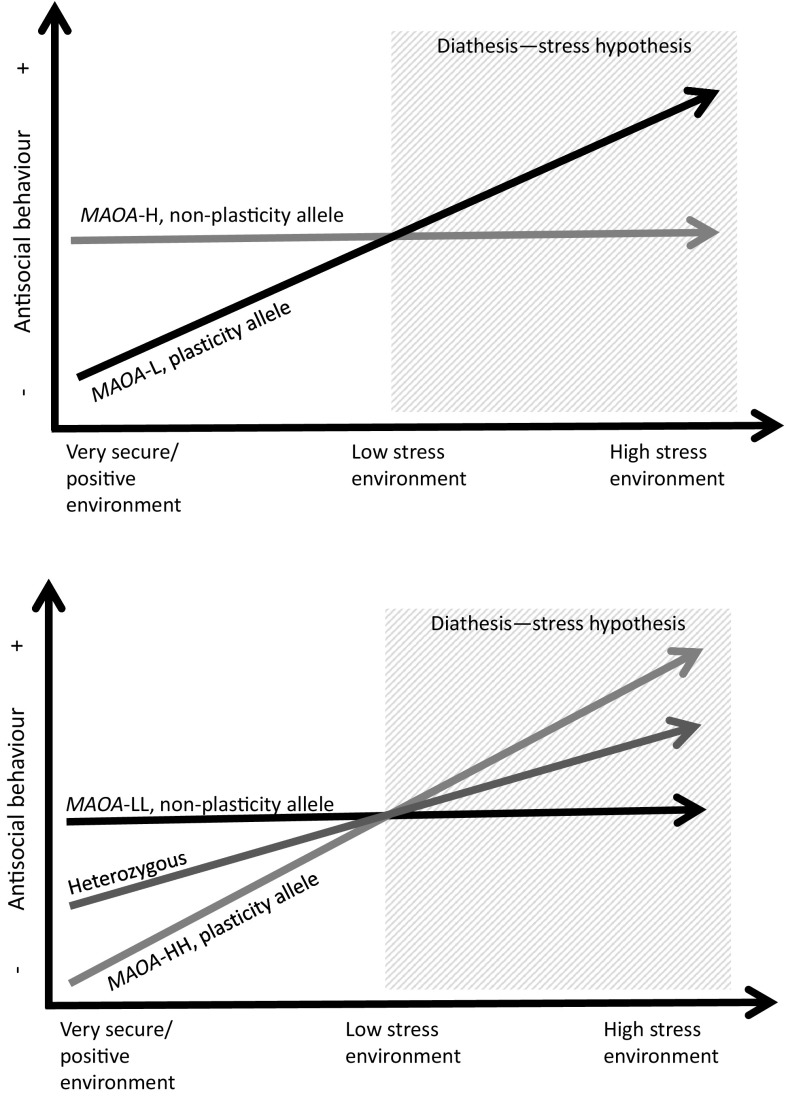
Contrasting the differential susceptibility hypothesis and the diathesis–stress hypothesis, and possible sex differences in *MAOA*-uVNTR in relation to ASB among males (**a**) and females (**b**)

In conclusion, the cumulative interpretation of findings described in the present review suggests possible differential susceptibility properties of *MAOA*-uVNTR. To advance the research field further, future studies should strive to investigate differential susceptibility effects of *MAOA*-uVNTR in relation to ASB by investigating statistically the G×E with both negative and positive environmental factors. The possible differential susceptibility properties as well as possible sex differences of *MAOA*-uVNTR in relation to ASB are illustrated in Fig. 1a, b.

### Implications of differential susceptibility effects

The theoretical assumption of possible plasticity properties of *MAOA*-L in males (i.e., lower risk for adverse outcomes in a positive environment and a higher risk for the same adverse outcome in those exposed to a negative environment) suggests that after adjustment for environmental adversity, the direction of the main effect of *MAOA* should vary depending on the psychosocial risk load of the sample population. Because the prevalence of maltreatment and adversity is low and has a positively skewed distribution in the general population, any statistically significant main effects are, therefore indicative of a lower risk for ASB in *MAOA*-L carriers among most males who have had little or no exposure to environmental adversity. Therefore, in statistical models, adjusting for environmental effects, such opposite main effects in the risk/plasticity allele in general population samples may be interpreted as indications of differential susceptibility properties of *MAOA*-uVNTR. Correspondingly, a psychosocial high-risk sample (e.g., psychiatric populations, prison inmates, demographically high-risk samples) with a high prevalence of environmental adversity, even though not measured in the statistical model, would be more likely to exhibit main effects of *MAOA*, in which *MAOA*-L would be associated with increased risk for a negative outcome in males [e.g., see Armstrong et al. (2014)].

In terms of sex differences, an unexpected similar main effect of *MAOA* has been reported in a female general population sample. In that study, the main effect of *MAOA*-HH was a lower risk for a negative outcome but a simultaneous higher risk for a negative outcome for the interaction of *MAOA*-HH with environmental adversity (Prom-Wormley et al. 2009). However, opposite *MAOA*-HH main effects, such as higher risk for an antisocial outcome (Sjoberg et al. 2007; Verhoeven et al. 2012; McGrath et al. 2012) or hyperactivity (Enoch et al. 2010) have been reported in female MAOA-HH carriers.

### Focus on statistical coefficients

Most studies show no main effect of *MAOA* genotype in univariable analyses; therefore, if not adjusted for all effects in the model, each coefficient reflects only a small, often non-significant, piece of the puzzle. It is also indicative that there is no main effect of the gene, i.e., an association with *MAOA* emerges only when the model is adjusted for the environment. However, interpreting separate univariable effects or two-way interactions by using only the coefficients might be misleading.

We suggest the use of a model-dependent realistic analysis (Hawking and Mlodinow 2010) to investigate the cG×E in relation to ASB and whether the direction of the cG×E varies depending on both sex and different environmental exposures (Nilsson et al. 2015; Aslund and Nilsson 2018). At present, there is no solid evidence for the possible phenotypical susceptibility properties of *MAOA* in relation to ASB based on the function of different *MAOA* alleles (cG×E), combinations of different susceptibility genes (cG×cG×E), or effects of different mixtures of positive and negative environmental exposures (cG×E-pos×E-neg) in different populations. Therefore, directional hypothesis-driven methods as an additive AGRS can be questioned, especially because the interactions might be subadditive (i.e., 2 + 2 = 3) or superadditive (i.e., 2 + 2 = 10) (Goldman and Rosser 2014).

Consequently, if the sex and genetic and environmental factors are truly interactive, a statistical model will constantly change depending on which factors and interaction terms are included in the analysis. Therefore, it is always important to include the relevant interaction terms in the applied models, but to also consider that the statistical model is a rough draft of a theoretical model, which in turn is a very simplified outline of a biological phenomenon within a complicated web of positive and negative environmental factors, as well as interacting genetic factors. Statistical models should therefore be seen as a tool—one of many other tools in the research arsenal—and secondary to a theory—knowledge of the literature and solid logical argument (Hayes and Rockwood 2017). As indicated in previous research (Nilsson et al. 2015; Aslund and Nilsson 2018), there might be a dose–response pattern of negative environmental exposure; even among those with experiences of both physical and sexual abuse, there is a protective effect of experiencing positive social relations.

Taking this standpoint may mean that the interpretation of main effects based on *p* values and correlation coefficients may be superseded because such main effects can change for each interaction term entered into the model. Similarly, an interpretation of a two-way cG×E-neg interaction may change if a significant cG×E-pos interaction is included in the model, and consequently, both the two-way interactions of E-neg and E-pos will change if there is a significant three-way interaction cG×E-neg×E-pos, and so forth. Therefore, the functional meaning of the results of statistical models must be inferred by the researchers who translate the results of the outputs (Hayes and Rockwood 2017).

Furthermore, sample size estimation has been a frequently debated topic ever since the critical review of the first 10 years of cG×E interaction research was presented (Duncan and Keller 2011). In their influential work, Duncan and Keller (2011) gave examples of sample sizes based on effect sizes calculated by equating the effect sizes found “for genetic main effects in large genome-wide association studies (GWAS), which provide the most reliable information about the true effect sizes of genetic main effects” (p 1044). The authors also suggested that “In sum, unless cG×E effect sizes are over an order of magnitude larger than the typical genetic main effect sizes detected in GWAS, then cG×E studies have generally been underpowered, perhaps severely so” (Duncan and Keller 2011, p 1044). This assumption might have been correct if there was such a thing as a main effect of a candidate gene associated with ASB. As we argue, if the theories of genetic plasticity are correct and implicate individual differential sensitivity to both E-pos and E-neg, the genetic main effect is irrelevant because it depends on the specific environmental factors that have been included in the model (Aslund and Nilsson 2018; Nilsson et al. 2015). Both E-pos and E-neg factors have a major predictor-intersection problem, perhaps best described as a problem with multicollinearity, which makes power calculations difficult. Others have argued that many of the best-designed studies for testing the cG×E hypothesis have samples < 300 and that these methods had better control over the estimations of the variables in the studies (Moffitt and Caspi 2014; Uher and McGuffin 2008; Caspi et al. 2010; Karg et al. 2011).

Sample size estimates have been shown to decrease as within-subject correlations increase. Moreover, the sample size needed to detect an effect size for a three-way interaction is four-fold that required to detect the same effect size for a two-way interaction (Heo and Leon 2010). On the other hand, in hypothesis testing science, the importance of sample size is secondary to more primary considerations of the quality of the measures and correctness of the design (Moffitt and Caspi 2014). Therefore, hypothesis-driven, well-designed studies might instead focus on interpreting the specific changes in the effect sizes because of the different interactions (Hayes and Rockwood 2017) rather than focusing on power calculations and excessive corrections for multiple testing. There is also ambiguity about the validity of the traditional causal steps approach to interaction tests, also referred to as mediation or moderation distinction (Baron and Kenny 1986), which has amid the lowest power among methods for testing for intervening variable effects. Consequently, the building of models that goes beyond the traditional step approach can be performed using modern methods of mediation analysis (Hayes 2009).

In our view, traditional layouts for uni- and multivariable analyses can, and perhaps should, be avoided. Rather, it would be more helpful if investigators tested the full, most realistic model of their sample with all possible interaction effects adjusting for different known confounding factors (Keller 2014). By doing so, another potential problem might be avoided, i.e., the need to correct the *p* value for multiple testing (Perneger 1998; Nakagawa 2004).

There are some important implications for the generalizability of most previously reported cG×E findings. First, the direction of the effect in the classical diathesis–stress model in a large community sample, which includes participants characterized by several risk and protective factors of varying intensity, would be expected to differ from the results of studies of high-risk samples who have been exposed to more adverse and fewer protective factors, based on the different environmental background load in the samples. Second, the absence of a negative environmental exposure is not equal to evidence of the presence of a positive environment. Third, previously reported findings in meta-analyses may be inconsistent because the analyses did not adjust for positive environmental factors. Consequently, the validity of future cG×E studies and meta-analyses would increase by considering both negative and positive environmental factors, and their interactions. Fourth, there is a vast predictor-intersection problem, e.g., postulating that the interaction between recent sexual abuse with *MAOA*-uVNTR is associated with ASB, whereas previous adolescent sexual abuse or childhood physical, emotional or other forms of abuse are not, is problematic. Therefore, future research needs to build models to adjust for the fact that a participant who is sexually or physically abused has probably experienced several other forms of negative life events and that these effects are cumulative. Fifth, there is an outcome-intersection problem, i.e., phenotypes vary depending on the characteristics of the population (age, sex and social and cultural characteristics). If there is a common unmeasured phenotype associated with several different outcome measures such as ASB, CD, ASPD, MD or anxiety, one would expect to find different results between males and females, in different age groups, in different populations and according to the location of the investigation. For this reason, careful statistical modelling is needed when using models in which the independent variables are associated with both each other and the outcome measure, and in which the effect on the outcome is dependent on the other predictor variables.
